# The Elephant and the Blind Men: Making Sense of PARP Inhibitors in Homologous Recombination Deficient Tumor Cells

**DOI:** 10.3389/fonc.2013.00228

**Published:** 2013-09-11

**Authors:** Silvana B. De Lorenzo, Anand G. Patel, Rachel M. Hurley, Scott H. Kaufmann

**Affiliations:** ^1^Division of Oncology Research, Mayo Clinic, Rochester, MN, USA; ^2^Department of Molecular Pharmacology and Experimental Therapeutics, Mayo Clinic, Rochester, MN, USA

**Keywords:** PARP inhibitor, synthetic lethality, non-homologous end joining, homologous recombination, BRCA1, BRCA2, ovarian cancer, breast cancer

## Abstract

Poly(ADP-ribose) polymerase 1 (PARP1) is an important component of the base excision repair (BER) pathway as well as a regulator of homologous recombination (HR) and non-homologous end-joining (NHEJ). Previous studies have demonstrated that treatment of HR-deficient cells with PARP inhibitors results in stalled and collapsed replication forks. Consequently, HR-deficient cells are extremely sensitive to PARP inhibitors. Several explanations have been advanced to explain this so-called synthetic lethality between HR deficiency and PARP inhibition: (i) reduction of BER activity leading to enhanced DNA double-strand breaks, which accumulate in the absence of HR; (ii) trapping of inhibited PARP1 at sites of DNA damage, which prevents access of other repair proteins; (iii) failure to initiate HR by poly(ADP-ribose) polymer-dependent BRCA1 recruitment; and (iv) activation of the NHEJ pathway, which selectively induces error-prone repair in HR-deficient cells. Here we review evidence regarding these various explanations for the ability of PARP inhibitors to selectively kill HR-deficient cancer cells and discuss their potential implications.

## Introduction

Poly(ADP-ribose) polymerase (PARP) inhibitors are currently undergoing extensive testing as potential anticancer agents (1–13). These drugs were initially developed as modulating agents that could enhance the cytotoxicity of DNA damaging treatments such as ionizing radiation and temozolomide (1, 12, 14). Interest in these agents was heightened by the demonstration that *BRCA1*- and *BRCA2*- (*BRCA1/2-*) mutant cancer cells are selectively killed by single-agent PARP inhibitor treatment (15, 16). Consistent with these preclinical observations, the PARP inhibitor olaparib has exhibited substantial single-agent activity in *BRCA1/2*-mutant breast and ovarian cancer (17–21). Nonetheless, fewer than 50% of patients with *BRCA1/2*-mutant cancers respond to these drugs, raising important questions about identifying patients most likely to derive benefit from PARP inhibition (22, 23). With this in mind, extensive efforts have been directed at further refining the mechanism of cytotoxicity of PARP inhibitors and elucidating mechanisms of resistance.

To provide a context for discussing the selective killing of BRCA1/2-deficient cells by PARP inhibitors, we first briefly outline what is known about the PARP family of enzymes and the repair of DNA double-strand breaks. We then describe and discuss four models that have been proposed to account for the selective killing of homologous recombination (HR)-deficient cells by PARP inhibitors.

## PARPs: A Family of ADP-Ribosyltransferases

The molecular biology and biochemistry of the PARP family of ADP-ribosyltransferases have been extensively reviewed elsewhere (24–33) and will only briefly be summarized here. Originally described in the 1960s (34–36), PARP1 is the founding member of a family of enzymes (37, 38) that transfer ADP-ribose moieties from the dinucleotide NAD^+^ to polypeptide acceptors, thereby catalyzing either mono- or poly(ADP-ribosyl)ation of polypeptide substrates (24, 39, 40). Although 18 members of the PARP family have been identified in mammalian cells (24, 25), only 6 are known to synthesize poly(ADP-ribose) polymers (1, 25, 41). Three of these family members, PARP1, PARP2, and PARP3, have been implicated in DNA repair (31). Of these, PARP1 is the most abundant (up to 10^6^ copies/nucleus) and has been shown to play critical roles in DNA repair, epigenetic modification of chromatin, regulation of genomic stability, modulation of cellular energy pools, the regulation of transcription, and a distinct form of cell death termed parthanatos (25–32, 42).

Although other PARPs might play an important role in the response to PARP inhibitors (43), existing models of PARP inhibitor-induced cytotoxicity emphasize the role of PARP1. Moreover, despite the well-established effects of PARP1 modulation on transcription (28), chromatin structure (26, 28, 44), and energy metabolism (1, 30, 33), current explanations for the lethality of PARP inhibition in HR-deficient cells focus solely on the role of PARP1 in DNA repair.

In response to certain types of DNA damage – particularly DNA nicks and double-strand breaks – PARP1 catalytic activity increases as much as 500-fold (41, 45, 46). This activation reflects a recently described conformational change that is transmitted from the DNA binding domains at the N-terminus of the PARP1 molecule through intervening domains to the catalytic domain at the C-terminus, resulting in altered alignment of critical residues in the active site (41, 47, 48). Once activated, PARP1 adds poly(ADP-ribose) moieties to a wide range of nuclear proteins, including histones, topoisomerases, and other non-histone chromatin proteins, although PARP1 itself is the major protein that is covalently modified (41, 49). The resulting poly(ADP-ribose) polymers not only alter the function of the covalently modified proteins (49–52), but also serve as a new binding site for other nuclear proteins (32, 41, 53–55).

Through this ability to synthesize poly(ADP-ribose) polymer, which covalently or non-covalently interacts with a variety of nuclear proteins, PARP1 contributes to a number of different steps in DNA damage response pathways. In its most extensively studied role, PARP1 is essential for base excision repair (BER) (56–58), a process involving the removal of a single damaged base and subsequent restoration of DNA integrity (59, 60). After recruitment to the damaged DNA, PARP1 recruits the scaffolding protein X-ray cross complementing protein 1 (XRCC1) (57, 61), which in turn binds to various BER proteins, bringing together a variety of components required for efficient repair of different base lesions (59, 62).

The involvement of PARP1 in DNA repair is not limited to XRCC1 recruitment during BER. PARP1 has also been reported to play a critical role in HR (63–65), including recruitment of MRE11 and NBS1 to DNA double-strand breaks (66), and to competitively inhibit the classical non-homologous end-joining (NHEJ) pathway by preventing Ku binding to free DNA ends (67). In addition, PARP1 plays a critical role in restarting replication forks that stall as a consequence of nucleotide depletion or collisions with bulky lesions (68–71). Any or all of these roles of PARP1 in DNA repair might be important in understanding the cellular effects of PARP inhibitors.

### Homologous recombination

**Figure 1:**
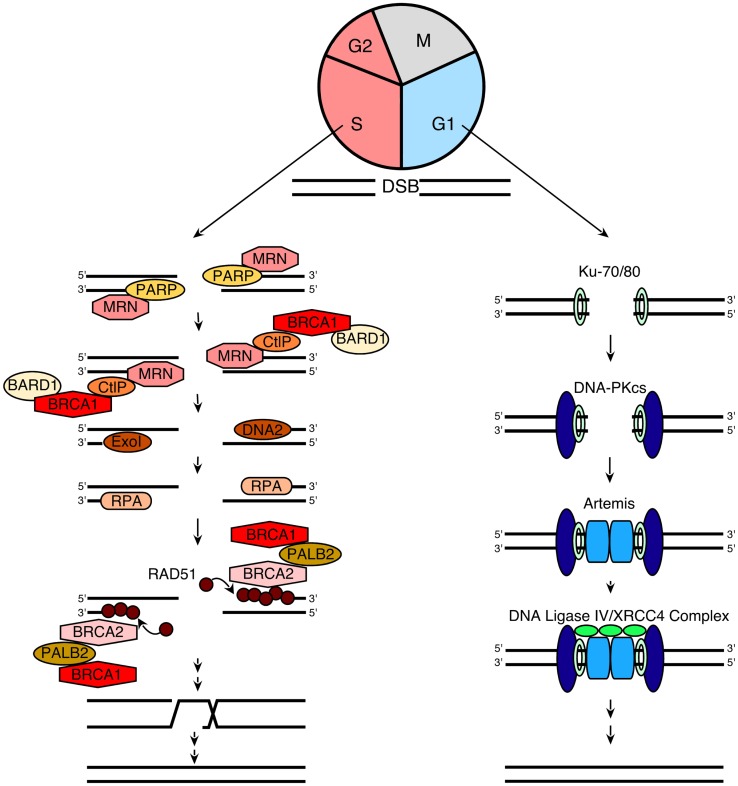
**A simplified model for NHEJ and HR**. When a DNA double-strand break (DSB) occurs during G1, it is repaired via NHEJ (right). This process involves the following steps: (1) the Ku70/80 heterodimer detects and binds to the DSB; (2) Ku70/80 bound to the DSB recruits DNA-PKcs; (3) DNA-PKcs undergoes autophosphorylation, favoring the processing of DNA ends by Artemis; and (4) the XRCC4/DNA ligase IV complex ligates the processed DNA ends. Additional details regarding NHEJ can be found in refs (109–111). In contrast, when a DSB occurs during the S and G2 phases of the cell cycle, repair occurs preferentially via the HR pathway (left), which involves the following steps: (1) PARP1 binds to the DSB (48) and competes with Ku binding to DNA ends (67); (2) the MRN complex is recruited (66) to the DSB (together with CtIP and BRCA1/BARD1) and mediates the initial stages of DSB resection; (3) extensive end resection is catalyzed by EXO1 and DNA2/BLM (79, 80), resulting in long stretches of ssDNA; (4) this ssDNA is coated by RPA; (5) the BRCA2/PALB2/BRCA1 complex facilitates replacement of RPA with Rad51 (73, 81); (6) RAD51 filaments induce strand invasion into homologous DNA sequences; (7) DNA polymerization occurs using the sister chromatid as a template; and (8) resolution of the resulting complexes produces an exact copy of the template where the DSB was generated. Additional details of the HR process can be found in Refs. (60, 72, 73).

In order to understand the models that currently describe the action of PARP inhibitors in HR-deficient cells, we also briefly review the process of HR itself. When DNA double-strand breaks form, two pathways compete to repair them (Figure 1): HR, which is a high fidelity pathway, and NHEJ, which is error-prone. According to current understanding (60, 72, 73), the HR pathway is activated when components of the MRN (MRE11/Rad50/Nbs1) complex bind to DNA double-strand breaks. In brief, Nbs1 brings its binding partners MRE11 and Rad50 to the nucleus, where the complex binds to double strand breaks (74). This MRN complex then recruits phosphorylated CtIP, which activates the exonuclease activity of MRE11 (75–78). After activated MRE11 resects one strand of the DNA to generate relatively short 3′ single-stranded DNA (ssDNA) tails, two different exonucleases, ExoI and DNA2, extend the single-stranded tails to a length of several thousand basepairs by continuing the resection (79, 80). The resulting ssDNA is rapidly bound by the ssDNA binding protein replication protein A (RPA), which is then replaced by Rad51 to form a nucleofilament as described in greater detail below. This Rad51-ssDNA complex facilitates homology searching and invasion of the ssDNA into homologous duplex DNA sequences of its sister chromatid. Once the resected ends are annealed to complementary strands, intervening sequence is synthesized using the intact strand as a template and ligated into place (81).

A critical step in the HR pathway is the loading of Rad51 onto ssDNA. This step is the culmination of a long series of reactions (Figure 1) that are triggered in response to DNA damage (72, 82). Once the MRN complex binds to DNA double-strand breaks, it also recruits and activates the DNA damage-activated kinase ATM, resulting in ATM autophosphorylation followed by sequential phosphorylation and recruitment of the histone variant H2AX, the “mediator” (scaffold) protein MDC-1, and several other proteins, including the tumor suppressor protein BRCA1, to sites of DNA damage (73, 82). Partner and localizer of BRCA2 (PALB2) binds to the C-terminus of BRCA1 and N-terminus of BRCA2, creating a bridge to recruit BRCA2 to sites of DNA damage. BRCA2 then binds phosphorylated Rad51, targeting active Rad51 to the ssDNA (83).

This entire HR process is tightly linked to cell cycle progression in multiple ways (84). First, BRCA2 and Rad51 are only expressed in S and G2 phases of the cell cycle, making HR impossible in G1 (76). Second, the cyclin-dependent kinase CDK2, which is active primarily at the G1/S transition and in S phase, catalyzes a priming phosphorylation of CtIP that is required before DNA damage can induce CtIP binding to MRN and subsequent MRE11-initiated end resection (85, 86). Finally, G0 and G1 cells have not replicated their DNA and, therefore, lack sister chromatids that provide homologous sequences for HR.

### HR deficiency defines certain malignancies

The complex HR process can be interrupted at any of a number of steps. In particular, HR fails to occur efficiently if genes encoding components of the MRN complex, CtIP, ATM, MDC-1, H2AX, PALB2, BRCA1, BRCA2, or Rad51 are silenced or mutated at critical residues. Mutations that disable these proteins, as well as other participants in the HR process, are often found in cancers (73). In high-grade serous ovarian cancer, for example, *BRCA1* and *BRCA2* mutations are found in roughly 15% of cases, with mutations in another dozen or more HR genes found in an additional 10–15% of cases (87–89). While some of these mutations are familial, as many as half appear to be sporadic (89, 90). These mutations and the resulting genomic instability are a hallmark of high-grade serous ovarian cancer (90). Likewise, mutations in *BRCA1, BRCA2, PALB2*, and other components with the HR pathway are common in familial and certain subtypes of sporadic breast cancer, particularly triple negative breast cancer (91–93). *PTEN* is deleted or silenced in over 50% of endometrial cancers and a substantial fraction of glioblastomas and prostate cancers (94–97).

Early studies found that BRCA1- or BRCA2-deficient cells are hypersensitive to PARP inhibitors (15, 16). In particular, cells lacking BRCA1 or BRCA2 were more susceptible to PARP inhibitor-induced apoptosis and showed more profound growth inhibition when treated as xenografts in nude mice (15, 16). Subsequent investigation demonstrated that cells deficient in other HR components, including NBS1, ATM, ATR, Chk1, Chk2, Rad51, Rad54, FANCD2, FANCA, PALB2, or FANCC, are also hypersensitive to PARP inhibitors (98–100). Moreover, cells lacking the lipid phosphatase PTEN were shown to be deficient in Rad51 expression (101, 102), also leading to PARP inhibitor sensitivity (102). Accordingly, the demonstration that PARP inhibitors are active, relatively non-toxic anticancer agents (17–21) led to substantial enthusiasm for developing these agents to treat a variety of neoplasms that exhibit HR deficiency.

Given the tantalizing preclinical and early clinical activity of PARP inhibitors in HR-deficient tumors, there has also been substantial interest in inducing a state of temporary HR deficiency in hopes of sensitizing cancers that lack inactivating mutations in the Fanconi anemia (FA)/HR pathway. Previous studies have demonstrated that this can be accomplished by treating cells with epidermal growth factor receptor inhibitors (103) or cyclin-dependent kinase inhibitors (104), which promote BRCA1 trafficking from the nucleus to the cytoplasm; phosphatidylinositol-3 kinase inhibitors, which downregulate Rad51 (105) or BRCA1 and BRCA2 (106); ATR inhibitors, which diminish replication stress-induced activation of cell cycle checkpoints and repair (107), or even possibly PARP inhibitors themselves (108). Whether this pharmacological inhibition of HR will sensitize cancer cells in the clinical setting as effectively as inactivating mutations in FA/HR pathway genes remains to be determined.

### NHEJ as an alternative mechanism of DNA repair

In addition to HR, which is a high fidelity repair process, cells also can employ the more error-prone NHEJ pathway to repair double-strand breaks. In essence, NHEJ is a process that detects free DNA ends, trims incompatible DNA, and directly ligates the double helix to restore DNA integrity (Figure 1). As reviewed elsewhere (109–111), this process involves initial binding of the Ku70/Ku80 heterodimer to free DNA ends, resulting in recruitment of the large serine/threonine kinase DNA-PKcs. Once bound to the DNA terminus, DNA-PKcs phosphorylates itself as well as a number of enzymes that can process DNA ends, including the nuclease Artemis, polynucleotide kinase phosphorylase, and DNA polymerases. Finally, the DNA ends are ligated by the DNA ligase IV/XRCC4 complex. Because cells in G1 lack both the DNA substrate and much of the protein machinery required for HR, NHEJ is the major pathway used for DNA double-strand break repair during G0 and G1. Moreover, this pathway is thought to play a major role in DNA repair when HR is impaired.

Previous studies have demonstrated that the NHEJ pathway is regulated in a number of ways. First, a complex containing the large scaffolding protein 53BP1 and its binding partner Rif1 inhibits accumulation of BRCA1 and the HR regulator CtIP at sites of DNA damage, thereby facilitating NHEJ in preference to HR (112–115). Second, ATM-mediated phosphorylation modulates the activity of the NHEJ nuclease Artemis (111). Third, Ku70, Ku80, and DNA-PKcs have all been previously identified as binding partners of poly(ADP-ribose) polymer (pADPr) (54, 57); and more recent studies suggest that other NHEJ components such as XRCC4 and Artemis also interact with pADPr (55). Additional studies have indicated that pADPr inhibits the NHEJ pathway, providing a starting point for one of the models describing the cytotoxicity of PARP inhibitors (15, 116).

### Choice between HR and NHEJ

Several factors determine whether a DNA double-strand break is repaired by HR or NHEJ (117, 118). The lack of BRCA2, Rad51, and a suitable sister chromatid as a template prevent HR during the G0 and G1 phases of the cell cycle. During S and G2 phases, on the other hand, there is a competition between HR and NHEJ. For example, Ku70 and Ku80 binding impairs double-strand break end resection, whereas resection prevents binding of the Ku70/Ku80 complex (119, 120). Additional studies have shown that MRN plays a primary role in removing or displacing Ku from DNA ends to allow resection to take place. When damage occurs during the G1 phase of the cell cycle, the 53BP1/Rif1 complex restricts CtIP recruitment and stimulation of MRE11-mediated resection as described above, thereby facilitating NHEJ (112–115). During the S and G2 phases of the cell cycle, on the other hand, Rif1 is inhibited by a BRCA1-CtIP complex, allowing HR to occur. These competing interactions illustrate the complexity of processes that regulate DNA repair and provide an explanation for the observation that mechanisms involved in DNA double-strand break repair shift from NHEJ to HR during S phase (121).

## Current Explanations for the Selective Cytotoxicity of PARP Inhibitors in HR-Deficient Cells

The seminal observation that PARP inhibitors selectively kill *BRCA1/2*-deficient cells in preclinical models (15, 16) was rapidly followed by the demonstration that PARP inhibitors exhibit clinical activity against *BRCA1/2*-mutant tumors (17–20). At least four different explanations have been advanced to explain this so-called synthetic lethality.

### BER inhibition

**Figure 2:**
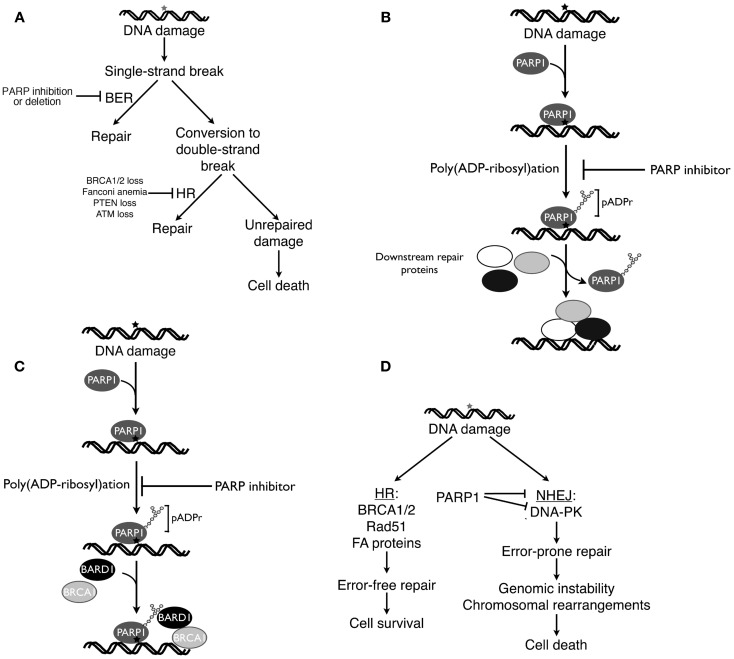
**Four current models of PARP inhibitor-induced cancer cell killing**. **(A)**, classical explanation of PARP inhibitor cytotoxicity in HR-deficient cells (124, 125). As described in the text, endogenous DNA damage is thought to result in DNA single-strand breaks, which ordinarily would be repaired by base excision repair (BER). If PARP inhibitors prevent BER, then persistent single-strand breaks are thought to be converted to DNA double-strand breaks, which would be repaired by HR in HR-proficient cells but remain unrepaired in HR-deficient cells. **(B)** Model emphasizing trapping of inhibited PARP1 at sites of DNA damage. According to this model, PARP1 binds to damaged DNA, synthesizes polymer, and then is released from the DNA so that repair enzymes can bind (51). Building on these observations, this model postulates that PARP inhibition results in failure of PARP1 to dissociate from sites of damage, leading to diminished access of other repair proteins, inhibited repair, and cell death. **(C)** Model emphasizing impaired recruitment of mutated BRCA1 in the presence of PARP inhibitors. As described by Li and Yu (134), recruitment of BRCA1 to DNA double-strand breaks requires both rapid binding of the BRCA1 binding partner BARD1 to pADPr and subsequent binding of a BRCA1-containing complex to phosphorylated H2AX at the break. Mutations that impair recruitment of the BRCA1-containing complex to phosphorylated H2AX render BRCA1 localization to sites of damage more dependent on the BARD1-pADPr interaction and, therefore, more sensitive to PARP inhibitors. **(D)**, model emphasizing the role of activated NHEJ in PARP inhibitor killing. When DNA double-strand breaks occur, HR preferentially repairs them. In HR-deficient cells, however, double-strand breaks are more frequently repaired by the error-prone NHEJ pathway, resulting in mutations, chromosomal rearrangements, and NHEJ-mediated cell death. PARP inhibitors accelerate this process by removing a brake on NHEJ (116). **(A,D)** are modified from Patel et al. (116).

Because PARP1 plays a critical role in BER (122, 123), initial explanations for the ability of PARP inhibitors to selectively kill HR-deficient cells focused on the interplay between BER and HR. According to this classical view [Figure 2A, see also Ref. (124, 125)], DNA damage induced by reactive oxygen species or replication errors results in DNA single-strand breaks, which ordinarily would be repaired by the BER pathway. Inhibition of PARP is postulated to cause persistence of these single-strand breaks, which are then converted to DNA double-strand breast as a consequence of interactions with transcription complexes and advancing replication forks. In HR proficient cells these DNA double-strand breaks would be repaired by HR. In the absence of BRCA1, BRCA2, or other HR components, however, impaired repair would result in persistence of these breaks and lethality. Accordingly, cells with fully active PARP1 or an intact HR pathway (*BRCA1/2* wild type cells) would be expected to survive these endogenous DNA insults, whereas cells with an HR defect treated with a PARP inhibitor would not (124, 125).

### Trapping of PARP1 at sites of DNA damage

An alternative model suggests that PARP1 becomes trapped on DNA in the presence of PARP inhibitors, thereby diminishing access of other repair proteins to damaged DNA. This model (Figure 2B) is based on some of the well-established characteristics of PARP1 reviewed above. In particular, PARP1 contains N-terminal zinc fingers that recognize damaged DNA, permitting PARP1 binding to various lesions (126), and increased pADPr synthesis (48, 127, 128). While PARP covalently modifies a wide range of substrates, most of the resulting pADPr is covalently bound to PARP1 itself (129), increasing the negative charge of the enzyme and eventually causing its dissociation from the DNA (51).

Studies performed over 20 years ago demonstrated that catalytically inactive PARP1, e.g., PARP1 lacking its substrate NAD^+^, inhibits DNA repair under cell-free conditions (51). Additional experiments showed that the DNA binding domain of PARP1, which is able to recognize damaged DNA but not catalyze pADPr formation, also acts as a dominant negative to enhance the cytotoxicity of certain DNA damaging treatments in intact cells (130, 131). PARP1 that has been catalytically inactivated by treatment with an effective small molecule inhibitor would likewise be expected to inhibit repair. This mechanism has recently been found to account for the ability of PARP inhibitors to enhance the cytotoxicity of the topoisomerase I poison topotecan (132) and the DNA methylating agent methylmethane sulfonate (MMS) (133). Extrapolating from these observations, it has been suggested that trapping of PARP1 at sites of endogenous DNA damage might account for the ability of PARP inhibitors to kill HR-deficient cells (Figure 2B).

### Defects in recruitment of BRCA1 to sites of DNA damage

Li and Yu recently reported that recruitment and retention of BRCA1 at sites of DNA damage reflects two different processes, (i) an initial interaction between poly(ADP-ribose) polymer at the damage site and the BRCT domain of the BRCA1 binding partner BARD1 and (ii) subsequent slower binding of a BRCA1-containing protein complex to phosphorylated histone H2AX at the damage site (134). Mutations that impair BARD1 interactions with poly(ADP-ribose) polymer, BARD1-BRCA1 complex formation, or binding of the BRCA1-containing protein complex to phosphorylated H2AX all reduce survival after DNA damage. Moreover, in the presence of PARP inhibitors, the initial rapid recruitment of the BARD1-BRCA1 complex to sites of DNA damage is impaired, making the cells more dependent on phospho-H2AX-mediated BRCA1 recruitment. Conversely, when mutations in the BRCT domain of BRCA1 impair participation of BRCA1 in the complex that interacts with phospho-H2AX, recruitment of BRCA1 to sites of DNA damage becomes dependent on poly(ADP-ribose)-mediated recruitment of BARD1 (134), providing another model to explain synthetic lethality between *BRCA1* mutations and PARP inhibitor treatment (Figure 2C).

### NHEJ activation

Although PARP1 clearly plays an important role in BER (14, 122), it is important to emphasize that PARP also regulates other repair processes (1, 30, 123, 135) as described above. Earlier observations suggested that a variety of DNA repair proteins, including Ku70, Ku80, and DNA-PKcs, can be regulated by ADP-ribosylation (135). In particular, Ku70, Ku80, DNA-PKcs, and more recently Artemis were identified as pADPr binding proteins (53–55). Moreover, the interactions of Ku70 and Ku80 with pADPr inhibit classical NHEJ (67, 136–138). These observations prompted several groups to examine the potential contribution of NHEJ pathway activation to PARP inhibitor-induced killing of HR-deficient cells.

Collectively, these studies have provided several pieces of evidence suggesting an important role for NHEJ activation in PARP inhibitor-induced killing. PARP inhibitor treatment results in DNA-PKcs activation in HR-deficient cells, as manifested by DNA-PKcs autophosphorylation and phosphorylation of the downstream substrate H2AX in a DNA-PK-dependent fashion (116). This PARP inhibitor-induced DNA-PKcs activation is accompanied by increased NHEJ activity as indicated by assays for repair of a plasmid that has a DNA double-strand break (116). Moreover, PARP inhibitors selectively induce chromosomal rearrangements and mutations in HR-deficient cells (15, 116). Importantly, this PARP inhibitor-induced increase in chromosomal rearrangements and mutations is diminished by simultaneous treatment of HR-deficient cells with a selective DNA-PK inhibitor (116). Likewise, the cytotoxicity of PARP inhibitors is diminished by manipulations that diminish NHEJ activation, including Ku80 siRNA (116), DNA-PKcs inhibition (116), or DNA-PKcs deficiency (116, 139, 140). Based on these results, a model for PARP inhibitor-induced cytotoxicity that emphasizes activation of the NHEJ pathway has been proposed (Figure 2D). In this model, some endogenous source of DNA damage results in DNA double-strand breaks. If cells are HR proficient, the HR pathway repairs this damage with high fidelity. If cells are HR deficient, however, then end resection-dependent NHEJ is activated (116) and contributes to error-prone repair that results in mutations and chromosomal rearrangements (Figure 2D).

Consistent with this model, deletion of 53BP1, which is required for NHEJ pathway activation, leads to PARP inhibitor resistance (141). Likewise, 53BP1 loss was shown to rescue the lethality of deleterious *BRCA1* mutation in mouse models (142, 143), suggesting that *BRCA1* deficiency kills mouse cells by activating NHEJ.

### The elephant and the blind men

Like the blind men examining the elephant, each of these models emphasizes a different aspect of PARP1 biology. Just as none of the blind men in the parable could provide a complete description of the elephant, we believe that the present models explain certain facets of PARP inhibitor-induced lethality but also leave some questions unanswered.

#### The role of poly(ADP-ribose) polymers in recruitment of BRCA1 to sites of DNA damage

The observations summarized in Figure 2C provide substantial new insight into the recruitment of BRCA1 to sites of DNA damage. Nonetheless, this model fails to explain PARP inhibitor sensitivity of HR-deficient cells in general. As the authors themselves point out, this model cannot explain the enhanced PARP inhibitor sensitivity of cells that totally lack BRCA1 (as opposed to expressing a BRCT domain mutant). Moreover, it is unclear how this model accounts for the synthetic lethality observed when cells lacking BRCA2, Rad51, or other downstream components of the FA/HR pathway are treated with PARP inhibitors (15, 98).

#### Trapping of PARP1 at sites of DNA damage

We are concerned that the model shown in Figure 2B also fails to account for critical observations regarding PARP inhibitor-induced killing. In particular, this model is a classical enzyme poisoning model, where the inhibited enzyme becomes an agent that contributes to cellular demise. This type of model, for example, accounts for the cytotoxicity of topoisomerase I poisons such as camptothecin (144). For this class of drugs, the poisoning model accounts for a number of critical observations: (i) loss of the target enzyme is not lethal (145, 146); and (ii) because the lethality results from the cytotoxic action of the inhibited enzyme rather than the inhibition of product production, the killing effect is observed at concentrations far below those that inhibit all activity of the enzyme (144). Importantly, this type of model accurately predicts that elevated expression of the target enzyme will increase the lethality of drugs that poison the enzyme and diminished expression of the target enzyme will decrease the lethality of the poisons (144).

Recent reports suggest that PARP inhibitors sensitize to certain DNA damaging agents by poisoning PARP1 (Figure 2B) as proposed by Lindahl and coworkers two decades ago (51). In particular, it has been reported that cells selected for resistance to the DNA methylating agent temozolomide in combination with the PARP inhibitor veliparib express markedly diminished levels of PARP1 (147). As the authors point out, this is difficult to explain if PARP inhibitors are sensitizing cells by diminishing total cellular levels of poly(ADP-ribose) polymer below a critical threshold (catalytic inhibition) but are readily understood by the poisoning model put forward in Figure 2B. Likewise, recent studies of topoisomerase I poison/PARP inhibitor combinations are also compatible with this type of PARP1 poisoning model (132). In particular, PARP1 downregulation or knockout abolishes the ability of the PARP1 inhibitor veliparib to sensitize cells to topotecan or camptothecin, establishing PARP1 as the critical target for this sensitization. Importantly, however, PARP1 knockdown or knockout does not result in cells that are hypersensitive to camptothecin or topotecan (132). Instead, *Parp1*^−/−^ cells and *Parp1*^+^*^/^*^+^ cells exhibit identical camptothecin sensitivity in the absence of PARP inhibitors (132), suggesting that PARP1 catalytic activity is not essential for camptothecin resistance. *Parp1* gene deletion likewise protects chicken DT40 cells from the methylating agent MMS in combination with PARP inhibitors without rendering the cells hypersensitive to MMS alone (133), suggesting that PARP1 catalytic activity is also not required for MMS resistance. Consistent with a poisoning model, further experiments examining the topoisomerase I poison/PARP inhibitor combination have shown that transfection of *Parp1*^−/−^cells with catalytically inactive PARP1 or the isolated PARP1 DNA binding domain sensitizes to camptothecin just like treating *Parp1*^+^*^/^*^+^ cells with a PARP inhibitor (132). Collectively, these observations suggest that trapping of inhibited PARP1 on damaged DNA, which has previously been reported to prevent access of repair complexes (51), contributes to the cytotoxicity of certain types of drug-induced DNA lesions (133, 147, 148) as illustrated in Figure 2B.

On the other hand, it is difficult to see how the poisoning model in Figure 2B can account for the synthetic lethality between HR deficiency and PARP inhibition. As described above, this type of model in which the inhibited enzyme is the lethal agent predicts that cells lacking PARP1 will be resistant to PARP inhibitors and cells containing elevated PARP1 levels will be hypersensitive. Contrary to this prediction, a number of groups have demonstrated that PARP1 downregulation kills BRCA1/2-deficient cells (15, 16, 116), suggesting that PARP inhibitors are killing *BRCA1/2*-deficient cells by diminishing the production of poly(ADP-ribose) polymer rather than trapping PARP1 at sites of DNA damage.

#### BER inhibition

In contrast to the preceding model, the classical model that focuses on the role of PARP1 in BER (Figure 2A) is consistent with the observation that PARP knockdown kills HR-deficient cells. It should also be acknowledged that this model provided part of the rationale for testing PARP inhibitors in BRCA2-deficient cells in the first place (16). Nonetheless, this model makes several predictions that have been difficult to verify experimentally.

First, the model predicts that DNA ss breaks will accumulate after PARP inhibition. Work by Helleday and coworkers, however, has demonstrated no induction of ss breaks by PARP inhibitors (149, 150). It is, of course, possible that the putative PARP inhibitor-induced ss breaks are converted to DNA double-strand breaks so rapidly that they are not detected. Further study of this issue, perhaps with more sensitive assays for DNA ss breaks, appears to be warranted.

A second issue relates to the reported effects of XRCC1 knockdown. If ss break repair is playing a critical role in the cytotoxicity of PARP inhibitors, then the effect of downregulating other ss break repair components such as the scaffolding protein XRCC1 immediately downstream of PARP1 (151) should recapitulate the effect of PARP1 downregulation. However, XRCC1 downregulation has no impact on survival of *BRCA2*-mutant PEO1 ovarian cancer cells, whereas PARP1 downregulation is cytotoxic (116). Importantly, the XRCC1 knockdown was sufficient to sensitize the cells to MMS, suggesting that BER had been inhibited. These results imply that PARP1 exerts a role outside of ss break repair in HR-deficient cells (116).

Collectively, these observations call into question the suggestion that PARP inhibitors are inducing so-called synthetic lethality in the setting of HR by inhibiting ss break repair. Further testing of additional predictions of the model shown in Figure 2A is clearly needed.

#### NHEJ activation

As indicated above, a number of observations suggest that NHEJ plays a critical role in PARP inhibitor-induced killing (15, 116, 139–141). The model shown in Figure 2D, which emphasizes the role of PARP in regulating NHEJ, is consistent with these observations. Nonetheless, a number of questions about this model also remain unanswered.

First, it is unclear whether all components of the NHEJ pathway contribute equally to PARP inhibitor sensitivity. Available studies only show what happens if 53BP1, Ku80, or DNA-PKcs is disabled. In view of observations that “atypical” NHEJ can occur in the absence of certain components (110), it remains to be determined whether loss of Artemis, XRCC4, ligase 4, or other NHEJ components has the same impact on PARP inhibitor sensitivity.

Second, the available data suggest that inhibiting the NHEJ pathway diminishes cytotoxicity of PARP inhibitors in HR-deficient cells. However, additional research is needed to determine how these cells survive and repair DNA double-strand breaks if HR and NHEJ are both disabled.

Third, preclinical and clinical studies have suggested that PARP inhibitors are particularly effective in tumors that have deleterious mutations in HR pathway genes such as *BRCA1* and *BRCA2*. In contrast, tumors such as triple negative breast cancer that have *BRCA1/2* gene methylation appear to be less sensitive. It is unclear whether this reflects incomplete inhibition of the HR pathway by methylation, or whether NHEJ pathway genes might also be methylated in these tumors, leading to a repair status similar to *BRCA2*-mutant cells in which NHEJ components have been downregulated.

Finally, the model summarized in Figure 2D fails to specify the source of DNA damage that activates the NHEJ pathway. Given the importance of this putative damage to PARP inhibitor-induced killing, this question clearly warrants further study.

#### Should the models be combined?

Like the blind men in the parable, perhaps we can better understand the true nature of the elephant by merging several incomplete pictures. For example, it has been suggested (150) that inhibition of ss break repair (Figure 2A) might generate the DNA double-strand breaks (Figure 2D) that activate NHEJ and contribute to the cytotoxicity of PARP inhibitors. This would certainly be consistent with some of the known roles of PARP1 in DNA repair described above. On the other hand, the failure of PARP inhibitors to increase DNA ss breaks (149), like the failure of XRCC1 downregulation to reproduce the effects of PARP1 downregulation in BRCA2-deficient cells (116), raises concern that the hybrid model might not adequately account for the DNA damage that contributes to NHEJ-mediated killing. Given the other roles of PARP1, e.g., in restarting stalled replication forks (68–71), it is equally plausible that PARP inhibitor-induced collapse of stalled replication forks or disruption of some other PARP1-mediated process provides the DNA double-strand breaks that trigger NHEJ. Clearly, like the blind men, we require additional information to generate a coherent picture.

## Translation to the Clinic: Why the Correct Mechanism Matters

In contrast to chronic myelogenous leukemia, where the vast majority of patients respond to a Bcr/Abl kinase inhibitor (152), or BRAF V600E-mutant melanoma, where the response to vemurafenib is also above 50% (153, 154), early studies have suggested that PARP inhibitors have only a 30–40% response rate in *BRCA1/2*-mutant ovarian and breast cancers (19–21). In an era of increasingly personalized cancer treatment, a less than 50% chance of responding to a supposedly tailored therapy is somewhat disconcerting (22). By understanding the mechanistic basis for the synthetic lethality between HR deficiency and PARP inhibition, it might be possible to better understand why some HR-deficient cancers respond and others do not.

The models described above make different predictions about the cancers most likely to benefit from PARP inhibitor therapy. For example, the poisoning model shown in Figure 2C predicts that HR-deficient tumors with elevated PARP1 levels should be hypersensitive to PARP inhibitors. In contrast, the models shown in Figures 2A,D, which emphasize catalytic inhibition of PARP1 as the triggering event, predict that HR-deficient tumors with lower PARP1 levels will, if all other factors are equal, be more sensitive to PARP1 inhibitors because they will require less drug to decrease poly(ADP-ribose) polymer levels below a critical threshold. The model shown in Figure 2D further predicts that HR-deficient cancers with diminished levels of NHEJ proteins will be relatively resistant to PARP inhibitors, whereas the model in Figure 2A predicts that HR-deficient cancers with diminished levels of NHEJ proteins will be more sensitive to PARP inhibitors because they are dependent on NHEJ for repair of DNA double-strand breaks in the absence of HR.

In order to understand why some HR-deficient cancers respond to PARP inhibitors and others do not, these predictions need to be tested in the clinic. In addition, it will also be important to assess the relationship between response and more classical determinants of drug sensitivity such as levels of the target enzyme PARP1 or drug uptake and efflux.

In order for these correlative studies to proceed, it will be important for patients enrolling in PARP inhibitor trials to undergo biopsies prior to drug treatment to determine the status of DNA repair pathway genes. Whenever possible, investigators are also encouraged to obtain additional biopsies at the time of progression in order to determine the properties of cells that have resisted PARP inhibitor treatment. In this way, future studies can potentially allow identification of patients most likely to benefit from PARP inhibitor treatment.

In summary, current models describing the mechanistic basis for selective killing of HR-deficient cells by PARP inhibitors emphasize different aspects of PARP1 biology. Just as the blind men needed more information to make sense of the elephant, we need additional information in order to understand the action of these promising new agents. Given the need to improve the therapeutic outcomes for patients with HR-deficient tumors such as high-grade serous ovarian cancer, as well as the tantalizing activity of PARP inhibitors in this setting, further preclinical and clinical efforts to understand this new class of agents appear to be warranted.

## Conflict of Interest Statement

The authors declare that the research was conducted in the absence of any commercial or financial relationships that could be construed as a potential conflict of interest.
